# British and Irish Hypertension Society response to ‘RAAS inhibitors in pregnancy, breastfeeding and women of childbearing potential: a review of national and international clinical practice guidelines’

**DOI:** 10.1038/s41371-025-01004-w

**Published:** 2025-04-24

**Authors:** Luca Faconti, Louise Abrams, Bernadette Jenner, Eduard Shantsila, Sarah Partridge, Ian B. Wilkinson

**Affiliations:** 1 King’s College London British Heart Foundation Centre, Department of Clinical Pharmacology, 4th Floor, North Wing, St. Thomas’ Hospital, Westminster Bridge, London, SE1 7EH UK; 2 Retired, at the time of writing Homerton University Hospital, Homerton Row, London, E9 6SR UK; 3 Division of Experimental Medicine, University of Cambridge, Addenbrooke’s Hospital, Cambridge, CB2 0QQ UK; 4 Department of Primary Care and Mental Health, University of Liverpool, Liverpool, L69 7ZX UK; 5 Brighton and Sussex Medical School, University of Sussex, Brighton, BN1 9PH UK

**Keywords:** Hypertension, Preventive medicine

## Abstract

In their review of clinical practice guidelines on the use of renin-angiotensin-aldosterone system (RAAS) inhibitors, *Greenlees and Delles* call for more explicit advice on the use of these drugs among women of childbearing potential, during pregnancy and breastfeeding. In response, the British and Irish Hypertension Society (BIHS) highlight the key issues for clinicians to consider when prescribing RAAS inhibitors to hypertensive women and suggest areas where further research is needed.

## Statement

The National Institute of Health and Care Excellence (NICE) hypertension guideline NG136 recommend renin-angiotensin-aldosterone system (RAAS) inhibitors as first-line antihypertensive treatment in both men and women aged under 55 years (except for those of Black African or African-Caribbean family origin), and those who have type 2 diabetes of any age and family origin. From Step 2 of the NICE treatment algorithm onwards, RAAS inhibitors are recommended to all people with hypertension [1].

The NICE hypertension guideline NG136, supported by the Medicines and Healthcare products Regulatory Agency, caution that angiotensin-converting-enzyme inhibitors (ACE-I) and angiotensin II receptor antagonists (ARB) *“should not be used in pregnant or breastfeeding women or women planning pregnancy unless absolutely necessary, in which case the potential risks and benefits should be discussed*” [1, 2]. This is based on evidence that RAAS inhibitors are associated with adverse fetal and neonatal outcomes [3, 4].

NICE offers no specific advice on the management of women with unplanned pregnancies while taking RAAS inhibitors.

The UK Teratology Information Service (UKTIS) advise that there is no strong evidence that exposure to ACE inhibitors in the first trimester is associated with congenital malformations in the infant, but a lack of data means that the congenital malformation risk following exposure to ARBs cannot currently be quantified [5, 6]. The UKTIS advises women who have used an ACE-I or ARB in the first trimester, that no extra monitoring for birth defects is required. Women using RAAS inhibitors in the 2nd and 3rd trimester of pregnancy should be closely monitored.

The BIHS believe it is important to open the conversation with clinicians about their use of RAAS inhibitors in hypertensive women of childbearing potential and highlight areas where further research is needed.

The BIHS offer the following advice to clinicians to facilitate conversations with hypertensive women of childbearing potential who are not currently planning a pregnancy or pregnant:

- Discuss women’s pregnancy plans. For women of childbearing potential who are not actively planning a pregnancy or pregnant, discuss the benefits and risks to the use of RAAS-inhibitors. Support women to make an informed decision. Document conversations.
- Advise women to use safe and effective contraception while taking RAAS inhibitors.
- Advise women who take RAAS inhibitors for hypertension to stop these drugs 2–3 months before planning a pregnancy. Consider use of amlodipine or nifedipine or labetalol or methyldopa as alternative antihypertensive drugs, plus folic acid.
- In the event of contraceptive failure, RAAS inhibitors should be stopped as soon as possible. Further advice and information leaflets can be found at www.uktis.org.
- A recent study by Kitt and colleagues demonstrated optimising blood pressure (BP) control in the first 2–3 months postpartum after a hypertensive pregnancy, was associated with improved cardiac remodelling [7]. This highlights the importance of establishing good BP control in the immediate postpartum period.
- There is a lack of robust evidence on both the concentration of RAAS inhibitors in breastmilk and the subsequent effect on newborns. The decision on when to re-introduce RAAS-inhibitors will include elevated maternal BP uncontrolled on other antihypertensive therapies, specific medical and pregnancy history indicating the use of RAAS is critical, and the health and prematurity of the infant [8, 9].

### Research questions

1. To quantify the risk of congenital malformation after exposure to RAAS inhibitors in the first trimester of pregnancy.
2. To determine the optimal drug combinations to use during hypertensive pregnancies.
3. To determine the concentration of different antihypertensive drugs in breastmilk and their effect on newborns.

## Data Availability

All data generated and analysed for the development of this statement are included in this published article.
